# The Father, Son and Cholix Toxin: The Third Member of the DT Group Mono-ADP-Ribosyltransferase Toxin Family

**DOI:** 10.3390/toxins7082757

**Published:** 2015-07-24

**Authors:** Miguel R. Lugo, A. Rod Merrill

**Affiliations:** Department of Molecular and Cellular Biology, University of Guelph, Guelph, ON N1G 2W1, Canada; E-Mail: mlugoa@gmail.com

**Keywords:** cholix toxin, elongation factor 2, ADP-ribosylation, inhibitor development, computational chemistry

## Abstract

The cholix toxin gene (chxA) was first identified in *V. cholerae* strains in 2007, and the protein was identified by bioinformatics analysis in 2008. It was identified as the third member of the diphtheria toxin group of mono-ADP-ribosyltransferase toxins along with *P. aeruginosa* exotoxin A and *C. diphtheriae* diphtheria toxin. Our group determined the structure of the full-length, three-domain cholix toxin at 2.1 Å and its *C*-terminal catalytic domain (cholix_c_) at 1.25 Å resolution. We showed that cholix toxin is specific for elongation factor 2 (diphthamide residue), similar to exotoxin A and diphtheria toxin. Cholix toxin possesses molecular features required for infection of eukaryotes by receptor-mediated endocytosis, translocation to the host cytoplasm and inhibition of protein synthesis. More recently, we also solved the structure of full-length cholix toxin in complex with NAD^+^ and proposed a new kinetic model for cholix enzyme activity. In addition, we have taken a computational approach that revealed some important properties of the NAD^+^-binding pocket at the residue level, including the role of crystallographic water molecules in the NAD^+^ substrate interaction. We developed a pharmacophore model of cholix toxin, which revealed a cationic feature in the side chain of cholix toxin active-site inhibitors that may determine the active pose. Notably, several recent reports have been published on the role of cholix toxin as a major virulence factor in *V. cholerae* (non-O1/O139 strains). Additionally, FitzGerald and coworkers prepared an immunotoxin constructed from domains II and III as a cancer treatment strategy to complement successful immunotoxins derived from *P. aeruginosa* exotoxin A.

## 1. Introduction

Cholera disease continues to be a global health problem with thousands of victims each year, often occurring as a result of epidemics [1]. This disease is normally linked to secreted toxins (e.g., cholera toxin) produced by the O1 and O139 strains of *Vibrio cholera*. However, it is known that other strains of *V. cholerae* also produce secreted toxins in their quest for food and colonization. In fact, a number of non-cholera toxin-producing strains of *V. cholerae* have been shown to display virulence, suggesting that other virulence factors participate in this organism’s pathogenesis [2]. In certain cases, these toxins may allow colonization by non-pathogenic bacteria in symbiotic relationships with aquatic animal species [3].

**Figure 1:**
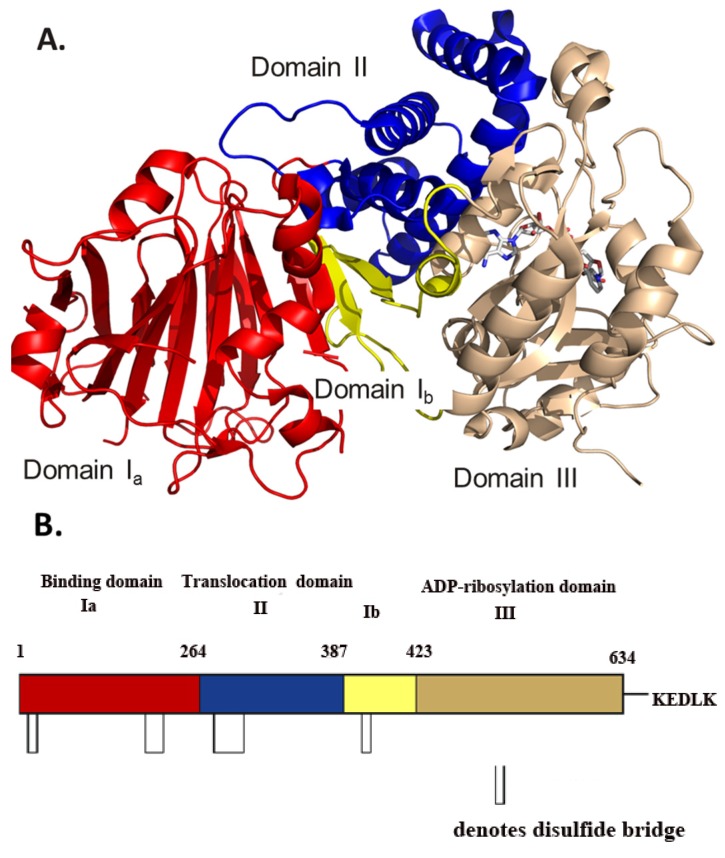
(**A**) Model of the 1.8 Å crystal structure of full-length cholix toxin in complex with NAD^+^. The cholix structure is shown as domain I_a_ (red), domain I_b_ (yellow), domain II (blue) and domain III (copper). The NAD^+^ moiety is shown in stick format with elemental colors. (**B**) A cartoon sequence of full-length cholix (leader sequence removed) is shown with the domains named and colored according to the scheme in (**A**) above. The approximate positions of the disulphide bridges are shown as open rectangles, and the *C*-terminal KDELK sequence is also shown (PDB: 3Q90).

Cholix toxin from *V. cholerae* was first identified in the *V. cholera* TP strain from aquatic samples in the U.S. [4]. Furthermore, a screen of *V. cholera* strains from both clinical and environmental sources revealed that the cholix *chxA* gene is present in nearly one-third of cholera environmental strains, being more common in non-O1/O139 strains [5]. Cholix toxin was recently characterized [6,7,8,9,10] and serves as a role model for structure-function characterization in the diphtheria toxin (DT) group of the mono-ADP-ribosyltransferase (mART) toxin family. Cholix is the third known member of the DT group within the mART toxin family, along with DT and *Pseudomonas aeruginosa* exotoxin A (ExoA). This family can be divided into two groups: CT (cholera toxin) and DT. Cholix is a 666-residue protein that possesses a signal peptide (residues 1–32) and a KDEL-like *C*-terminal sequence (KDELK), previously shown to be important for retrograde transport within the host cell endoplasmic reticulum [11]. The mature form of cholix is 71 kDa and contains four disulphide bonds. Although the cell biology for the intoxication process has not been well studied, it is likely that cholix follows a similar process in host cells to ExoA [12]. It is highly similar to ExoA (the catalytic domain III only) in three-dimensional structure, enzyme activity, target and inhibitor specificity [6,8]. However, despite the primary sequence similarity of domain III between cholix and ExoA toxins, the GC content of the *chxA* gene is different from genes found in *P. aeruginosa* (ExoA), suggesting that *chxA* was not the product of horizontal transfer between *P. aeruginosa* and *V. cholerae* [13]. Cholix is an A/B toxin that is comprised of a receptor-binding domain that is recognized by the low-density lipoprotein receptor-related protein (LRP-receptor), a membrane translocation domain for crossing the host cell membrane into the cytoplasm, and the enzymatic domain (Figure 1) [6].

**Figure 2:**
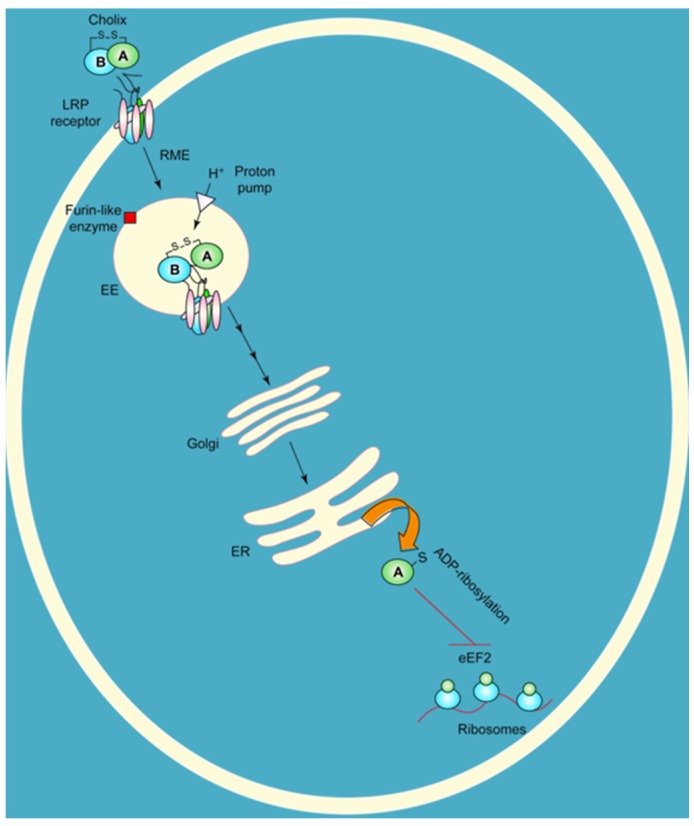
Target cell intoxication route of cholix toxin based on the known mechanism for *P. aeruginosa* exotoxin A. Cholix toxin binds to the LRP receptor protein on target eukaryotic cells, is internalized by receptor-mediate endocytosis (RME) and then is nicked to form the A and B fragments. The A fragment of cholix follows the retrograde pathway to the ER, where it is thought to translocate into the cytoplasm; it inhibits protein synthesis by modifying the diphthamide residue of eEF2 through its ADP-ribosylation activity. A furin-like enzyme is an endogenous protease that is believed to be responsible for nicking cholix in its Arg-rich loop during its intoxication mechanism.

*V. cholerae* is an aquatic organism that can often be found attached to the exoskeletons of crustaceans, and this behaviour may provide nutrients and protection against environmental stresses [14,15]. This organism may employ cholix as a tool to facilitate the ability of *V. cholerae* to colonize aquatic species, since cholix shows activity against eukaryotic cells by inhibiting protein synthesis in both mammals and crustaceans [6]. Cholix was also shown to be extremely toxic to yeast cells when expressed in the cytoplasm under the control of a copper-inducible system [8,16]. In this assay, it was shown that wild-type cholix caused a severe growth defect phenotype in yeast, whereas the catalytic signature variants, E574A and E581A, showed a significant recovery in the growth defect phenotype and a full recovery with the double variant, E574A/E581A [16]. Additionally, a yeast mutant of the elongation factor 2 (eEF2) target protein, G701R, conferred resistance to cholix, as well as to DT and ExoA and demonstrated that eEF2 is the cellular target for cholix in eukaryotic cells [16]. Cholix enters eukaryotic cells by receptor-mediated endocytosis through the LRP receptor in a similar fashion to ExoA [6]. It was suggested based on structural similarity to ExoA that activation of cholix occurs by reduction of a disulphide bond and cleavage by a furin-like protease in the endosome of the host cell [6]. The newly-formed catalytic fragment (residues 293–634) enters the cytoplasm and modifies eEF2 with ADP-ribose at the unusual diphthamide residue [17] (Figure 2). This reaction involves the transfer of ADP-ribose from NAD^+^ to the diphthamide residue on eEF2, leading to inhibition of protein synthesis and host cell death [6]. This reaction catalysed by DT group members has been well studied [18,19,20] and involves cleavage of the glycosidic bond (C–N) between nicotinamide and its linked ribose, as well as transfer of ADP-ribose to the imidazole group of the eEF2 diphthamide residue [20]. It was shown that a highly flexible loop 1 in cholix forms a solvent cover or water gasket to exclude the aqueous solvent from the reaction center and helps to stabilize the transition state for the reaction [20].

## 2. Cholix Toxin as a Virulence Factor in *V. cholerae*

Purdy *et al.* [5] assessed the prevalence of the *chxA* gene among a collection of 155 diverse *V. cholera*e environmental strains that originated in various countries, including Bangladesh and Mexico. It was found that nearly one-half of 83 non-O1 and non-O139 strains and nearly 20% of 72 O1/O139 strains harboured the chxA gene. It was concluded from this study that cholix plays a major role in the fitness of *V. cholerae* and that it may promote parasitic or mutualistic associations with aquatic dwelling eukaryotes. It may also enhance virulence during inflammatory gastrointestinal diseases in humans by combining with other virulence genes carried by non-O1 and non-O139 strains in which *chxA* is most prevalent. Although a role for cholix in causing diseases in man has not been clearly proven, at least two reports document diarrhea outbreaks in Peru and Kenya caused by non-O1/O139 *V. cholerae* isolates that were positive for cholix toxin [21,22]. In another study, the occurrence and genetic diversity of the *chxA* gene in both clinical and environmental *V. cholerae* strains that belong to O1 and O139, as well as non-O1/non-O139 serogroups were investigated. Three chxA toxinotypes were identified and correlated with their varied virulence patterns. Two toxinotypes, chxA I and chxA II, can cause extensive damage to the internal organs, especially the liver, of mice. Notably, the occurrence of the *chxA* gene among *V. cholerae* strains was found to be mostly independent of the presence of other major virulence genes. This observation along with the presence of the *chxA* gene among widely divergent non-O1 and non-O139 strains isolated from both patients and the environment highlights the potential of cholix toxin as an important virulence factor in non-O1/non-O139 strains of *V. cholera* [23].

**Figure 3:**
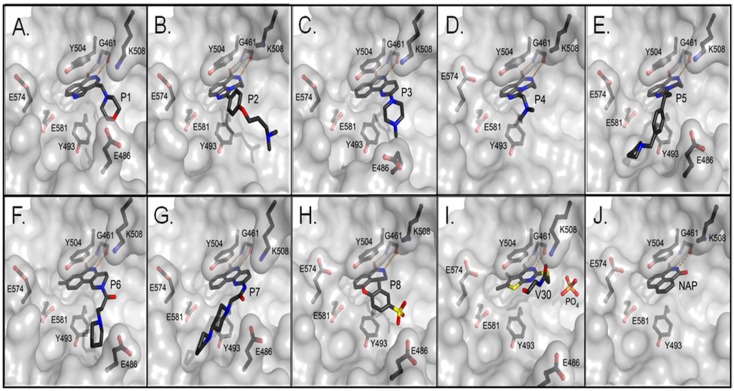
Crystal structures of the catalytic fragment of cholix_c_ bound to inhibitor compounds. (**A**) cholix-P1 (PDB: 3KI0), (**B**) cholix-P2 (PDB: 3KI1), (**C**) cholix-P3, (PDB: 3KI2), (**D**) cholix-P4, (PDB: 3KI3), (**E**) cholix-P5, (PDB: 3KI4), (**F**) cholix-P6, (PDB: 3KI5), (**G**) cholix-P7, (PDB: 3KI6), (**H**) cholix-P8, (PDB: 3KI7), (**I**) cholix-V30, (PDB: 3NY6), and (**J**) cholix-NAP (PDB: 3ESS). The inhibitors and nearby residues are shown in grey and black sticks, respectively. Hydrogen bonds are shown in orange dashed lines. All of the structures are from the Protein Data Bank with the PDB ID codes shown in parentheses.

## 3. Cholix Structure

Cholix is a useful model for ExoA and DT structure and function, because it is more amenable to crystallization; the three-dimensional structures of the catalytic domains of all three DT group toxins are highly superimposable and catalyse the same reaction with NAD^+^ and eEF2 as substrates. A total of 13 crystal structures of cholix have now been reported by our group, including the full-length cholix_FL_, 2.1 Å apo and 1.8 Å NAD^+^ structures [6,7], and several structures of the catalytic domain, cholix_c_, in complex with active-site inhibitors of the enzyme activity [6,8,16] (Figure 3). The cholix_FL_ structure consists of three domains that are quite similar to ExoA in structure, but not in sequence (only domain III shows significant sequence identity between these two toxins, 37%). These domains include a receptor-binding domain (domain Ia, 1–264; domain Ib, 387–423) that forms a 13-stranded antiparallel β-jellyroll, a translocation domain (domain II, 265–386) that is comprised of a bundle of six α-helices and a catalytic domain (domain III, 424–634) with an α/β fold topology (Figure 1). It is clear that domain III has the conserved ADP-ribosyltransferase (ADPRT) sequence pattern [24] and possesses a classic ADPRT fold domain (SCOP d.166.1.1, CATH 3.90.175.10) [25].

A furin-specific cleavage site protrudes from the surface of domain III (R^287^-S-R-K-P-R^292^) that is readily available for nicking by a furin-like protease [26]. The superposition of cholix onto the ExoA structure shows an RMSD of 2.04 Å for the C_α_ atoms with 1.70, 1.58 and 1.26 Å RMSD for domains I, II and III, respectively [6]. The positions of critical disulphides within the cholix structure align with those in ExoA. These include: Cys^11^–Cys^15^, Cys^208^–Cys^225^, C^278^–Cys^300^ and Cys^394^–Cys^400^.

**Figure 4:**
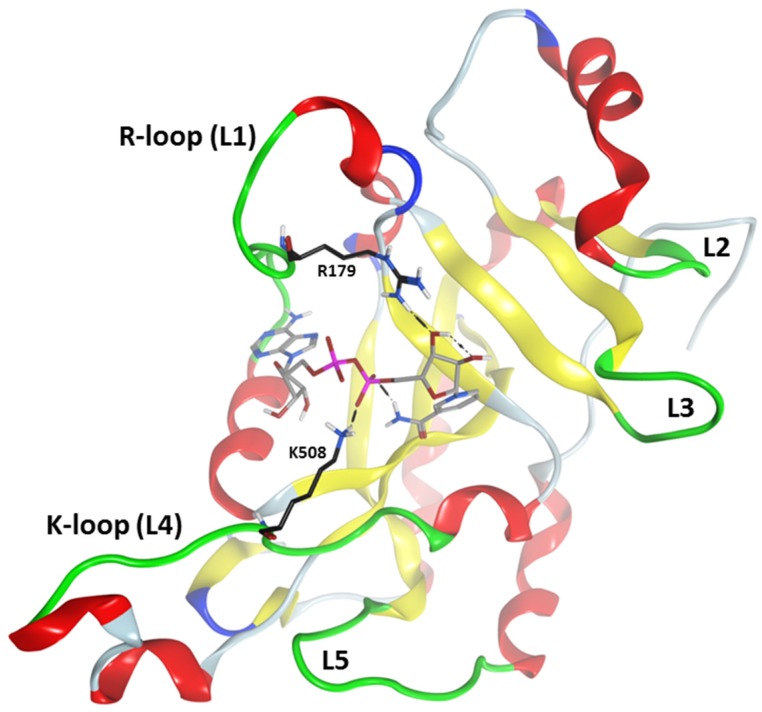
Active-site loops in cholix. Ribbon representation of cholix_c_-NAD^+^ complex showing the active-site loops highlighted in green. The substrate binding (L1–L4) and target recognition (L4–L5) loops are shown. The L-nomenclature corresponds to the loop definition in exotoxin A (ExoA) [19]; for cholix, the active-site loops include: L1, Arg^471^–Thr^483^; L2, Thr^544^–Pro^547^; L3, Glu^574^–Glu^579^; L4, Gly^503^–Gly^512^; and L5, Gly^601^–Asp^610^. The *C*-atoms for Arg^479^ and Lys^508^ are depicted in black, and L1 and L4 are renamed in cholix as the R- and K-loops, respectively. Bound NAD^+^ is shown in grey *C*-atoms.

A comparison between the cholix apo and NAD^+^-bound structures did not show substantial changes in the backbone trajectory (RMSD of 0.33 Å for C_α_), implying that NAD^+^ binding to cholix_FL_ did not trigger loop movements [4]. In contrast, the flexible loop 1 (L1: residues 458–463) is closed in the full-length ExoA, sterically blocks substrate binding and opens upon activation of the active-site structure (Figure 4) [27]. Indeed, the backbone structure of L1 residues along with the side chains of Arg^458^, Gln^460^ and Leu^462^ sterically prevent NAD^+^ binding. Thus, cholix can bind NAD^+^ in the absence of structural activation, although the cholix_FL_ shows only weak catalytic activity compared to the recombinant catalytic fragment, cholix_c_ (see the later discussion). Interestingly, the C_α_ distance map of the apo cholix_FL_ structure revealed that the region in the catalytic domain corresponding to the R-loop (residues: 471–483, L1 in ExoA) is one of the main contact loops with domain II, along with the K-loop (residues: 503–512, L4 in ExoA) [9,10] (Figure 4); therefore, the dynamic properties of this loop for cholix in solution must account for important conformational changes required in its proposed role as an active-site gate. Indeed, a quantitative assessment on the backbone motion showed relatively high mobility for these active-site loops [9].

In regards to the cholix catalytic domain, crystal structures either uncomplexed or bound with NAD^+^ remain elusive, while 11 X-ray complexes with various bound inhibitors revealed a lack of atomic coordinates for the R-loop; hence, computational modeling was necessary to assess the structural and dynamic properties of cholix [10]. In this sense, an *in silico* catalytic domain obtained by truncation of the cholix_FL_ X-ray structure was employed as a starting structure in molecular dynamics simulation work, yielding a root mean square fluctuation profile comparable to the empirical calculation of backbone dynamics. The enhanced mobility of cholix_c_, in comparison to the equivalent part of the cholix_FL_ structure relies on the lack of inter-domain contacts [9]. Surprisingly, the estimated mobility of the cholix K-loop “in solution” was higher than the R-loop (for both cholix_c_ and cholix_FL_), which is contrary to the behavior “in crystals”, although compatible with its shifted location when compared with the full-length set (two structures) with the C-domain set (11 structures). Notably, L4 in ExoA provides the association with domain IV of its eEF2 target [19], by interacting with an α-helix proximal to the modified residues, Dip^699^ on eEF2 (yeast protein; Dip^715^ for mammalian eEF2), the target of the ADP-ribose moiety. Interestingly, L4 remains close to Tyr^481^ in ExoA, which influences both the GH and ADPRT activities. Hence, the structure-encoded high mobility of the K/L4 loop in cholix_c_/ExoA is concordant with its function in target protein recognition or binding.

## 4. NAD^+^ Binding

**Figure 5:**
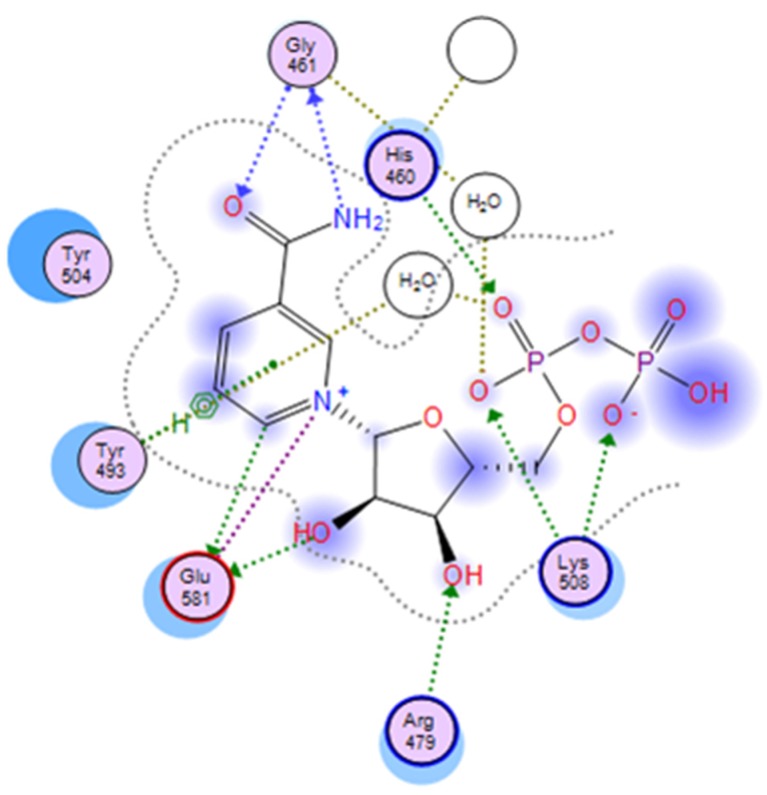
A two-dimensional interaction map of cholix_c_ with its bound NAD^+^. A 2D-representation of the PO_4_-PO_4_-ribose-nicotinamide part of NAD^+^ is shown in the active site of cholix_c_. Crystallographic water molecules interacting with pocket residues are indicated (H-bonding with side-chains or backbone atoms are shown as green or blue arrows, respectively). The H-bond bridging with water molecules is shown as gold-dotted lines, and salt bridges are shown as purple lines.

In solution, the NAD^+^ conformational distribution is complex, because it can access a large conformational space due to the high number of rotatable bonds and intra-molecular contacts. However, NAD^+^ usually adopts a rather compact conformation in solution and a more extended conformation when bound to proteins. Bound to toxins, NAD^+^ conformation within their active sites has been extensively examined [28]. ADP-ribosyltransferases, such as cholix, cleave the NAD^+^ *N*-glycosidic bond and bind NAD^+^ with several possible adenine mononucleotide conformations. In fact, a comparison of a number of the mART toxin-NAD^+^ complexes reveals that the adenine orientation varies more than the rest of the NAD^+^ molecule [7]. The adenine orientation must not be crucial to the enzyme function, giving rise to such variation. However, variation in the adenine ring is commonly observed within the CT group toxins, but not for the DT group. Interestingly, in the cholix-NAD^+^ structure, the NAD^+^ adenine ring is rotated as compared to DT and ExoA (Figure 5). It has been suggested that most mART toxin structures reveal a strained *N*-glycosidic bond to promote cleavage of the nicotinamide. For cholix-NAD^+^, the torsional angle (χ_N_ dihedral defined by the atoms O^4D^−C^1D^−N^1N^–C^2N^) and length of the glycosidic bond for bound NAD^+^ are quite typical and do not support the idea of a highly strained *N*-glycosidic bond in the Michaelis complex [7].

The binding pocket of NAD is tight around the nicotinamide and adenine moieties, but wider and more exposed in the middle section, at the ribose-phosphate-phosphate-ribose linker. In the cholix_FL_-NAD^+^ crystal structure, 35 pocket residues, a glycerol and five crystallographic water (CW) molecules are part of the recognition surface, offering a rather complex interplay of non-bonded interactions (Figure 5). These events include electrostatic interactions with the negative pyrophosphate and the positive nicotinamide, hydrogen bonding between the carboxamide and *N*-ribose hydroxyl and π-stacking with the nicotinamide and adenine rings. The key residues Gly^461^, Tyr^493^, Tyr^504^ and Glu^581^ at the N-site, along with the ribose-phosphate-phosphate-ribose part of NAD^+^ strongly interact with Arg^479^ (at the R-loop), Lys^508^ (at the K-loop) and His^460^, in addition to Ile^470^, Val^477^ and Gln^356^ at the A-site, and present the highest interaction energy (Figure 4). Accordingly, the ribose-nicotinamide portion of the NAD^+^ molecule interacts more strongly with cholix residues than the ribose-adenine moiety, which dictates that the nicotinamide half of the molecule is mainly responsible for driving the energetics of NAD^+^ binding to the cholix active site [9]. The three-dimensional reference-interaction-site model (3D-RISM) solvent analysis showed that NAD^+^ binding does not alter the water organization, since the five in-pocket CW molecules are also present in the apo form. However, the binding of NAD^+^ destabilizes these CW molecules, making them potentially displaceable by H-donor/acceptor ligand decorations (Figure 5). An exception is HOH^702^ (PDB: 2Q9O), which appears to increase the binding energy by bridging the substrate to Gly^461^ [9].

## 5. Kinetic Model and Substrate-Binding Properties

Cholix toxin shows both strong glycohydrolase (GH) and transferase (ADPRT) activities as a member of the DT group [6] (Table 1). Although cholix_FL_ only shows weak catalytic activity in the absence of activation, cholix_c_ (208 residues, 23 kDa) shows strong GH (*K*_M_, 67 µM; *k*_cat_, 1.92 h^−1^) and ADPRT (*K*_M_, 45 µM; *k*_cat_ 10 s^−1^) activities (Table 1) [6], serving as a model of the fully-active cholix enzyme. In spite of the observation that the cholix_FL_-NAD^+^ X-ray structure shows a single bound NAD^+^ molecule, for cholix_c_, both (i) a biphasic NAD^+^ binding isotherm as monitored by Trp quenching and (ii) substrate inhibition of the ADPRT activity using the analog *ε-*NAD^+^ was recorded, suggesting the coexistence of two bound substrate molecules [7]. These observations are consistent with our previous report for ExoA showing a biphasic binding curve for NAD^+^ constituents, such as AMP and ADP [29]. Furthermore, the observation of dual NAD^+^ substrates binding to cholix_c_ appears to be variant dependent, where the variants E574A, E581A and E574A/E581A did not appear to bind a second NAD^+^ molecule, while the variants Y493A, Y504A/F and E579R exhibited the wild-type behavior. Accordingly, a minimum kinetic scheme was proposed that suggests a random binding of NAD^+^ and eEF2, with thermodynamics (dissociation constants, *K*_d_’s; and interaction factors, *α*_i_’s), kinetics (catalytic rate constants, *k*_i_’s) and fluorescence (fractional quantum yields, *γ*_i_’s) parameters that account for the differential substrate curve of the fluorescence quenching and ADPRT activity among cholix_c_ wild-type and catalytic variants [7].

**Table 1:** Kinetic parameters of diphthamide-specific ADPRTs.

Parameter ^a^	ExoA_c_	DTA_c_	Cholix_c_
*K*_M (NAD)_ (µM)	121 ± 21	11 ± 1	45 ± 3
*V*_max_ (M s^−1^)	1.3 × 10^−7^	5.2 × 10^−6^	1.0 × 10^−7^
*k*_cat_ (s^−1^)	13 ± 2	5 ± 0.2	10 ± 0.5
^b^ *k*_cat_ (s^−1^) catalytic Glu→Ala	0.008 ± 0.0001	0.016 ± 0.002	0.004 ± 0.0003
*k*_cat_*/K*_M_ (M^−1^s^−1^)	1.1 × 10^5^	4.5 × 10^5^	2.2 × 10^5^

^a^ The kinetic parameters were determined under identical conditions for all three diphtheria toxin (DT) family members. The values represent the mean ± SD from three independent experiments. ^b^ The catalytic Glu to Ala mutations involved Glu^553^ (ExoA_c_), Glu^148^ (DTA_c_) and Glu^581^ (cholix_c_).

In an attempt to reconcile the cholix kinetic data with the structural information, a pocket searching algorithm over apo cholix_FL_ reported two subcavities connected to the main NAD^+^ site. The 3D-RISM solvent analysis predicted a significant hydrophobic character (densities) of these subpockets [9]. Clearly, one site is occupied by a glycerol molecule in the cholix_FL_-NAD^+^ structure, while the other site is filled with ethylene glycol in the apo cholix_FL_. This latter subpocket is exposed and close to the N-site. In fact, the extended structure of the P5 and P7 inhibitors shows their terminal moieties occupying this subsite in the respective cholix_c_-P5 and cholix_c_-P7 complexes.

Furthermore, the more compact P1 and P8 inhibitors showed a biphasic binding isotherm with cholix_c_, implying the coexistence of two bound molecules [8]. These results suggest that “effective” mutations involve the cholix_c_ N-site and indicate that the aforementioned subpocket might be part of the interaction surface for a second NAD^+^ molecule. If this is indeed the case, then the dynamic character of cholix_c_ (particularly the R-loop mobility) (Figure 4 and Figure 5) might offer the required flexibility to contact a NAD^+^ molecule partially bound at this subsite, since cholix_FL_ does not exhibit this biphasic behavior. Nevertheless, the physiological relevance of this second binding site is unknown so far.

## 6. Cholix Inhibitors and Pharmacophore Model

**Figure 6:**
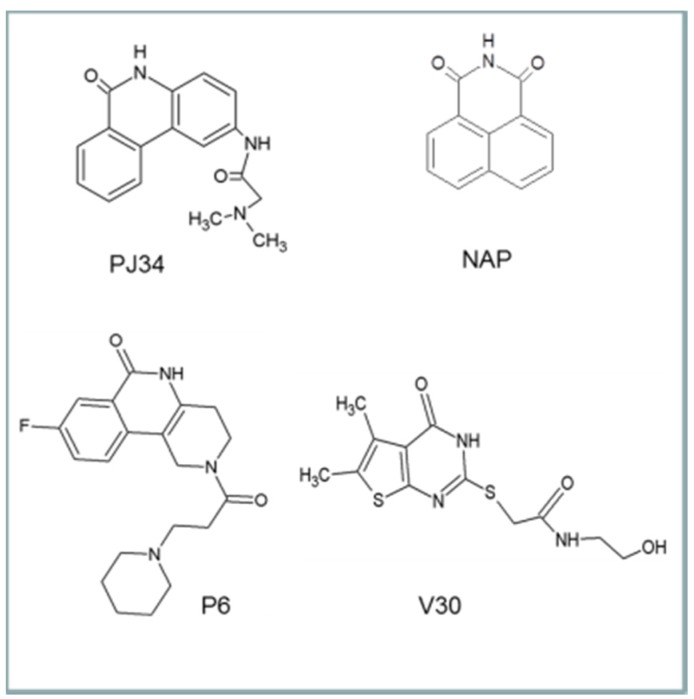
The four inhibitor platforms effective against cholix toxin. (1) Water-soluble phenanthridinones, such as PJ34; (2) naphthalimide derivatives, such as NAP, are more nonpolar than PJ34; (3) PARP-related inhibitors, such as P6, that are heterocyclics with a variety of substituents; (4) V30-related compounds; V30 was identified from a virtual screen against cholix toxin.

The first inhibitor developed for cholix was based on the finding that in the presence of a known PARP inhibitor, PJ34, cholix_c_ produced well-formed and highly diffracting protein crystals [6], while the cholix_c_-apo produced only poor quality crystals. Later, methods were devised to replace PJ34 with various inhibitors that were competitive for the nicotinamide moiety of NAD^+^ [8,16]. The inhibitors were drawn largely from two libraries of compounds. The first was a small library of known PARP inhibitors, and the second was a library of commercially available compounds generated from a virtual screen based on the 1.25 Å cholix_c_-PJ34 structure (PDB: 2Q6M). The key features for most cholix active-site inhibitors include a benzamido group fused into a hetero-ring structure, which mimics the nicotinamide moiety of NAD^+^ [30]. There are four main inhibitor platforms (Figure 6), one consisting of a water-soluble phenanthridinone backbone (PJ34), and the second is a naphthalimide derivative that is more nonpolar (NAP). The third platform (e.g., P6) is based on PARP heterocyclic drugs, and the fourth (V30) is based on the virtual screen experiment of 500,000 compounds from a commercial library [8].

In all inhibitor compounds, a key interaction is the H-bonding between the cyclic amide of the inhibitor and the backbone groups of a Gly residue within the Y-H-G conserved motif within active-site region 1 for DT group toxins [8,16]. Importantly, since the DT group toxins bind NAD^+^ in a different conformation than dehydrogenases and most NAD^+^-binding enzymes, the inhibitor libraries show reduced cellular toxicity towards the host eukaryote [8,31]. It was shown that PJ34 does not protect cells from toxin-induced cell death, likely because of its high water solubility, whereas NAP, inhibitors V30 (virtual screen) and P6 (and other directed PARP library compounds) show excellent efficacy (EC_50_ values: 170 nM–4.5 µM) in cell-based assays [8].

**Figure 7:**
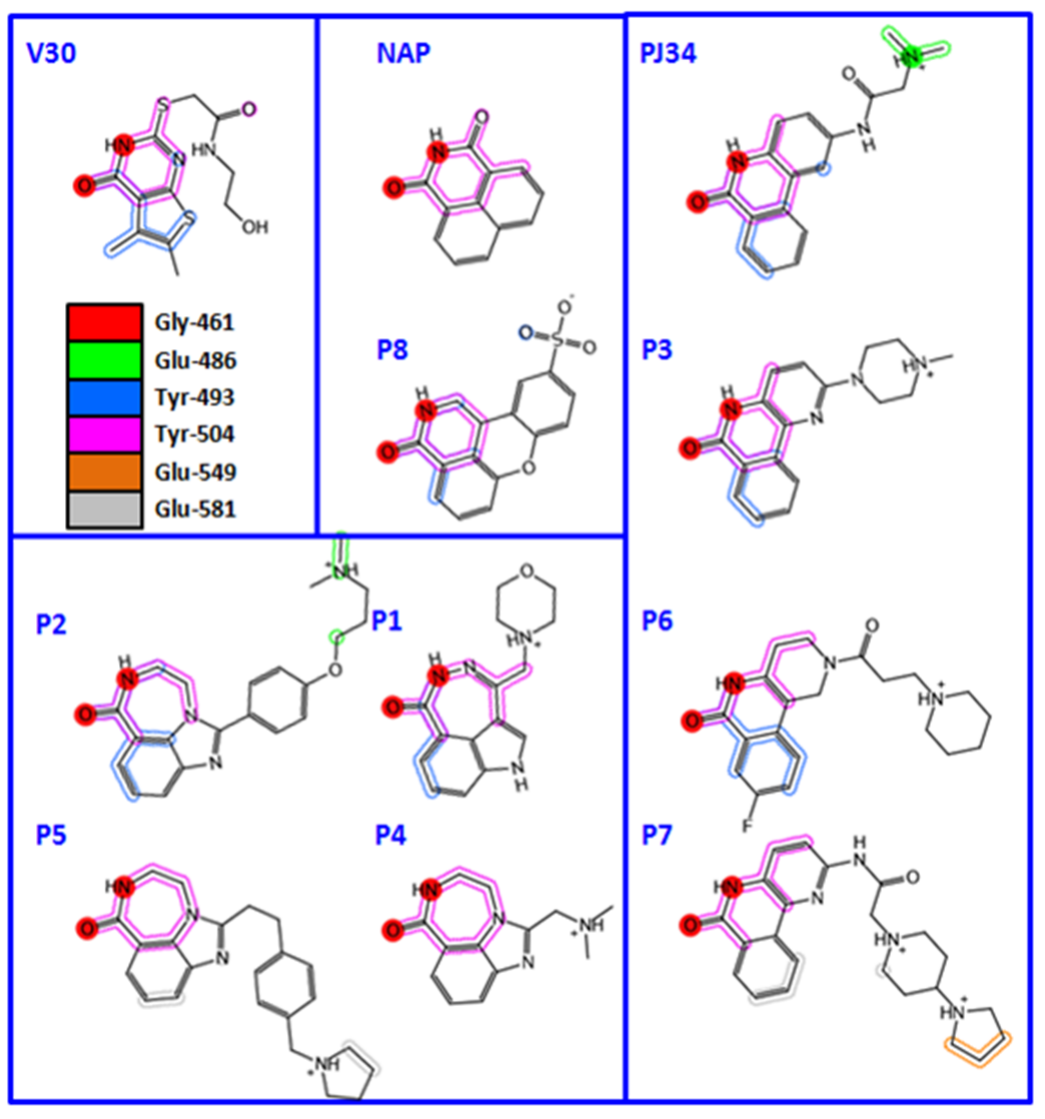
The 2D topological representations of V30, PJ34 and P-series inhibitor binding poses with cholix_c_. The colored sections/moieties represent the interaction with the pocket residues shown in the legend. The two red circles show the common interaction with Gly^461^. The 11 compounds were grouped according to common features of the inhibitor core region.

**Figure 8:**
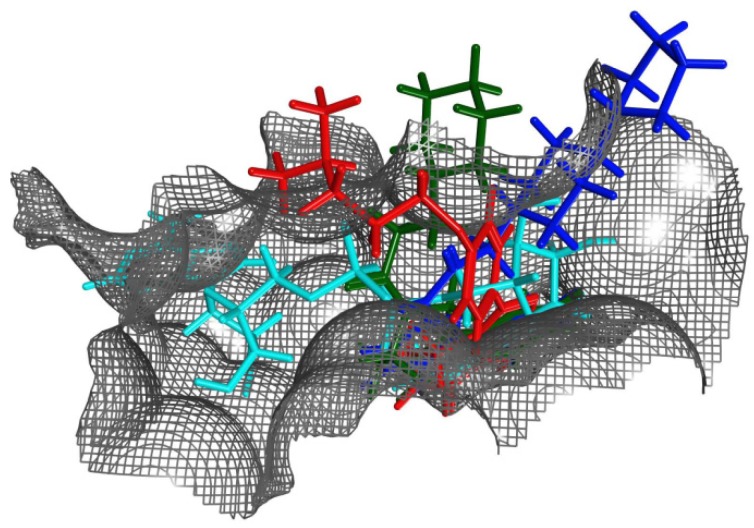
Ligands within the binding pocket of cholix_c_. Superposition of the binding poses (after optimal overlapping of the pocket Cα atoms) of NAD^+^ (cyan atoms) with representative inhibitors of the three classes of binding mode according to the spatial location of the terminal positive charge: P2 (red atoms), P6 (green atoms) and P7 (blue atoms).

The binding modes (topology and energetic) of these inhibitors were addressed by using 11 high-resolution (better than 1.65 Å) X-ray structures of cholix_c_ complexed with PJ34 [6], NAP [16], V30 and the P1–P8 (P-series) compounds [8] (Figure 7). In 10 of 11 cholix-inhibitor complexes (all but V30), the inhibitor “core” superposes three pharmacophoric features of the nicotinamide moiety of NAD^+^ (*i.e.*, H-donor/acceptor by the amide group and an aromatic center by the pyridinium ring), in addition to a common hydrophobic-center definition that emerges at the N-site. However, the “side-chains” of these inhibitors do not share any other pharmacophore character of NAD^+^ (e.g., the adenine aromatic centers) (Figure 8); rather, eight inhibitors with a positive charge match their cationic features in defined locations, regardless of the core structure, attachment point or chemical group of the inhibitor “tail” [10].

The conformational distribution of the flexible R-loop in cholix_c_, along with constitutive subpockets, would dictate the accessibility and energetics of these locations (Figure 8). Indeed, a 3D-quantitative structure-activity relationship (3D-QSAR) analysis demonstrated that the binding energy is modelled mainly from differential interactions of Arg^479^ (R-loop), Lys^508^ (K-loop), Glu^581^ and Gly^461^ (core), among others [10]. The case of inhibitor V30 is unique, since several differences between this compound and other inhibitor platforms are evident; however, the most obvious difference can be attributed to the absence of the fused benzamide as part of the core structure. Thus, the cholix_c_-V30 complex (PDB: 3NY6) presents features, such as (i) a rotated core ring-system in comparison to the NAD^+^ nicotinamide, (ii) out-of-plane atoms from two methyl groups into the flat N-subpocket and, more importantly, (iii) a strong interaction between a sulfur tail-atom and the core-residue Gly^461^ by a “sigma-hole” bond [32]. Currently, we are working to characterize several V30-derivatives based on the core structure of the inhibitor [33].

## 7. Cholix Induces Host Cell Apoptosis

Cholix induces host cell death through the use of different apoptotic pathways depending on the cell type. Cholix was found to induce cell death in HeLa cells involving multiple apoptotic pathways, with caspase-1, -4 and -5 being responsible for the early-stage caspase-dependent apoptosis [34]. It was shown that Bak/Bax knockdown in HeLa cells significantly suppressed cholix-induced cytochrome c release and caspase-7 activation. However, the activation of caspases-3 and -9 was observed in Bak/Bax knockdown HeLa cells, as well as in control cells. Overall, the findings suggest that the cholix-induced apoptotic pathway uses both the mitochondria-dependent and -independent pathways [34]. It remains unclear which factors are required between the modification of eEF2 by cholix and the activation of caspases by cholix-induced apoptosis. Mcl-1 and other short-lived anti-apoptotic proteins are believed to be the apoptotic trigger in cholix-treated cells. It was proposed that cholix-induced apoptotic pathways in HeLa cells occurs as follows: (i) cholix-induced inhibition of protein synthesis initiates a stress response, which starts a caspase-dependent apoptotic cascade through inflammatory caspase activation during early-stage apoptosis; (ii) subsequently, caspases induce both mitochondria-dependent and -independent apoptotic pathways; (iii) the mitochondria-dependent pathway is started by Bak/Bax assembly, followed by the release of cytochrome c and activation of caspase-7; (iv) following caspase-3 and -7 activation, these two apoptotic signaling pathways lead to HeLa cell death [34].

## 8. Cholix Toxin as an Immunotoxin for Cancer Treatment

The gene region encoding domains II and III of cholix (CET40) was cloned into an expression vector routinely employed to produce ExoA-based immunotoxins [35]. It was demonstrated that CET40 is a potent cytotoxic protein that can be targeted using an antibody to antigens on the surface of cancer cells. It was also shown that cell binding is mediated through the targeting antibody Fv and not by CET40 residues. Additionally, the immunological similarities of CET40 were compared with ExoA (PE40); the latter has produced a high rate of complete remissions in hairy cell leukemia and objective responses in other malignancies [36]. This is important because of the potential for using immunotoxins sequentially. Although PE40 and CET40 show high sequence homology and structural similarity, there was little immunological cross-reactivity shown between these two immunotoxins [35]. This implies that the major neutralizing epitopes of PE40 are not common with CET40, making it highly plausible to devise a strategy whereby PE40 immunotoxins are administered first, followed by a treatment with CET40 immunotoxins using the same targeting antibody. This treatment strategy has the potential to allow one or two additional cycles of immunotoxin cancer treatment.

## 9. Conclusions

Cholix toxin has emerged as a major virulence factor of non-cholera-producing strains of *V. cholerae.* It likely provides these strains with the ability to colonize the exoskeletons of crustaceans and related aquatic animals to provide the bacteria with a more stable food supply. The role of cholix toxin as a virulence factor leading to infection in humans has not yet been clearly established, although a role of cholix toxin in outbreaks in Kenya and Peru has not been ruled out. As the third member of the DT group of mART toxins, cholix now serves as a flagship for structure-function activities. The ability to readily obtain high-resolution crystal structures in the presence of substrates and inhibitors has provided powerful new insights into the mechanism of this notorious toxin family. Computational approaches have provided additional insights into the mART kinetic mechanism, and a pharmacophore model has been built to serve as the basis for the development of therapeutics for the treatment of infections caused by mART-producing pathogens. Finally, an immunotoxin consisting of cholix domains II and III has been prepared as an alternate treatment strategy in tandem with ExoA from *P. aeruginosa* for the treatment of certain human cancers.
